# Association analyses of the *INSIG2 *polymorphism in the obesity and cholesterol levels of Korean populations

**DOI:** 10.1186/1471-2350-10-96

**Published:** 2009-09-22

**Authors:** Seongwon Cha, Imhoi Koo, Sun Mi Choi, Byung Lae Park, Kil Soo Kim, Jae-Ryong Kim, Hyoung Doo Shin, Jong Yeol Kim

**Affiliations:** 1 Division of Constitutional Medicine Research, Korea Institute of Oriental Medicine, 483 Exporo, Yuseong-gu, Daejeon, 305-811, Republic of Korea; 2 Department of Genetic Epidemiology, SNP Genetics Inc., 14th Floor, Woolim-rall'ey B, Gasan-dong, Geumcheon-gu, Seoul, 153-803, Republic of Korea; 3 Obesity Clinic, Kirin Oriental Medical Hospital, 38-25 Jamwon-dong, Seocho-gu, Seoul, 137-905, Republic of Korea; 4 Department of Biochemistry and Molecular Biology, College of Medicine, Yeungnam University, 317-1 Daemyung-Dong, Daegu, 705-717, Republic of Korea; 5 Department of Life Science, Sogang University, 1 Shinsu-dong, Mapo-gu, Seoul, 121-742, Republic of Korea

## Abstract

**Background:**

While *INSIG2 *has been reported to be associated with BMI in many populations, conflicting results have prevented consensus over its role. In analyses of mice and cell cultures the gene has been found to be involved in the regulation of cholesterol synthesis; however, no relationship has been found with cholesterol metabolism in human epidemiological research. Therefore, this study attempts to assess the effect of rs7566605 near *INSIG2 *on both obesity- and cholesterol-related traits in Koreans.

**Methods:**

The rs7566605 polymorphism was genotyped with 2,364 Koreans, and associations with obesity- and cholesterol-related traits were analyzed statistically via an ANOVA or T-test.

**Results:**

Replication of an association with BMI, WHR, fat mass, fat percent, and abdominal fat area failed, and the C allele of rs7566605 was not associated significantly with total cholesterol, HDL cholesterol, or triglyceride. However, it was found in a meta-analysis of a dominant model that the C allele of rs7566605 appeared to affect the level of the total cholesterol, especially in female subjects.

**Conclusion:**

We failed to show associations of rs7566605 with cholesterol- and obesity-related phenotypes, although we newly suggest the possible involvement of *INSIG2 *with the plasma level of the total cholesterol in women.

## Background

*INSIG2 *is considered to be a candidate gene with respect to involvement in the development of obesity. A common variant located 10 kb upstream of the gene, rs7566605, was found to be associated with BMI in a recent genome-wide association study [1]. This association has been suitably replicated in several white [1-3], African-American [1], and Asian populations [4-6]. However, the SNP did not have a genetic effect on obesity according to other studies including white [2,7-14], Afro-Caribbean [12], and Asian populations [15-17]. Hence, the *INSIG2 *polymorphism may have an important effect in overweight populations under certain environmental circumstances, given several positive associations found in studies of overweight subjects [3,4].

INSIG2 has attracted the attention of researchers due to its role in cholesterol metabolism [18]. The protein is known to reside in the endoplasmic reticulum, where it binds to SCAP to inhibit it from convoying SREBPs to the Golgi apparatus [18]. Eventually, INSIG2 prevents SREBP from activating cholesterol synthesis because SREBP cannot be processed and activated by the Golgi enzymes. These actions of INSIG2 were also reported in subsequent research involving mice [18,19]. However, they have not been confirmed in research with human populations [4,6,8,12,13,15,20], although the prevalence of hypercholesterolemia was shown to be reduced in CC homozygotes of rs7566605 in Japanese-American women [16]. Even in that study, the SNP was not found to be associated with the levels of total cholesterol, HDL cholesterol, or triacylglycerol.

In the present study, a replication analysis for the association of rs7566605 with BMI as well as other obesity-related measures was conducted using Korean populations, as controversial results have also been reported with Asian populations. The genetic effects of *INSIG2 *rs7566605 on the cholesterol metabolic traits were evaluated in efforts to discover a possible role in human cholesterol metabolism.

## Methods

### Subjects

The Korean study population consisted of 996 subjects recruited from an obesity clinic at the Kirin Oriental Medical Hospital (Seoul, Korea) and 737 subjects recruited from an obesity clinic at the Yeungnam University Medical Center (Daegu, Korea) [21,22]. The 996 subjects from the Kirin obesity clinic were chosen from 1,302 individuals who came for help for weight control between 2001 and 2004. These subjects did not have chronic diseases, such as hypertension, coronary artery disease, stroke, diabetes, and hyperlipidemia. The 737 healthy unrelated subjects from Daegu, Korea were randomly recruited from an unselected population that came for a routine health check-up at Yeungnam University Hospital. These subjects were not representative of the Korean general population, as they had visited obesity clinics in order to address weight issues and/or as they had been the healthy selected subjects. In order to confirm this association, we recruited 631 subjects from 12 oriental medical hospitals in Korea (this population is hereafter named Multicenter) for 2 years since 2007, in which 266 patients with chronic diseases, like hypertension, hyperlipidemia, diabetes, and stroke were removed from a total of 897 individuals. All subjects provided written informed consent, and this study was approved by the Institutional Review Board of the Korea Institute of Oriental Medicine (for Kirin and Multicenter) and Yeungnam University. With the subjects from the Kirin Oriental Medical Hospital, additional anthropometric features (in this case the fat mass and fat percent) and abdominal fat mass areas were measured. Anthropometric features were measured via bio-impedance analysis using a commercial device (Inbody 2.0 Biospace, Korea). Abdominal fat areas were measured from computerized tomography cross-sectional images (Hispeed CT/e, GE, USA) as previously described [23,24]. Blood samples from all study subjects were drawn in the morning after overnight fasting. They were checked for biochemical measures of glucose, total cholesterol, triglyceride, and HDL cholesterol. They were also used for DNA extraction. The characteristics of the recruited individuals are listed in Table 1.

**Table 1:** Clinical characteristics of the enrolled subjects from two obesity clinics and Multicenter hospitals

**Feature**	**n (female %)**	**All**	**Male**	**Female**
**Kirin**				
Age (y)	996(93)	27.9 ± 8.43	29.3 ± 12.1	27.8 ± 8.07
BMI (Kg/m^2^)	996(93)	26.4 ± 4.70	32.6 ± 5.48	25.9 ± 4.27
Total cholesterol (mmol/L)	908(93)	4.92 ± 1.59	4.69 ± 1.41	4.93 ± 1.60
HDL cholesterol (mmol/L)	782(92)	1.60 ± 0.910	1.63 ± 1.29	1.59 ± 0.868
Triglyceride (mmol/L)	916(93)	1.01 ± 0.602	1.66 ± 1.09	0.963 ± 0.516
Glucose (mmol/L)	894(93)	5.63 ± 1.69	6.57 ± 2.81	5.57 ± 1.56
**Yeungnam**				
Age (y)	737(37)	45.5 ± 11.0	43.3 ± 10.6	49.0 ± 10.8
BMI (Kg/m^2^)	737(37)	27.1 ± 3.44	27.4 ± 30.6	26.5 ± 3.93
Total cholesterol (mmol/L)	737(37)	5.36 ± 0.960	5.38 ± 0.955	5.32 ± 0.968
HDL cholesterol (mmol/L)	737(37)	1.37 ± 0.346	1.31 ± 0.329	1.48 ± 0.345
Triglyceride (mmol/L)	737(37)	1.81 ± 1.39	2.04 ± 1.53	1.41 ± 1.01
Glucose (mmol/L)	736(37)	5.38 ± 1.36	5.47 ± 1.57	5.21 ± 0.903
**Multicenter**				
Age (y)	631(64)	43.4 ± 14.0	44.0 ± 13.6	42.1 ± 14.7
BMI (Kg/m^2^)	627(64)	23.0 ± 3.16	22.7 ± 3.24	23.4 ± 2.99
Total cholesterol (mmol/L)	600(64)	4.82 ± 0.809	4.84 ± 0.807	4.78 ± 0.815
HDL cholesterol (mmol/L)	601(64)	0.882 ± 0.0666	0.883 ± 0.0747	0.881 ± 0.0489
Triglyceride (mmol/L)	600(64)	1.31 ± 0.900	1.13 ± 0.677	1.64 ± 1.13
Glucose (mmol/L)	600(64)	5.15 ± 0.895	5.10 ± 0.803	5.25 ± 1.04

Mean ± standard deviation values are given.

### Genotyping

The rs7566605 SNP of the *INSIG2 *gene was genotyped with TaqMan [25], according to the manufacturer's instructions (Applied Biosystems, Foster City, CA). The intensities of the fluorescence in the assay products were read with Prism 7900 HT instrument (Applied Biosystems). The genotyping failed in 8 individuals of the Kirin and Yeungnam populations, therefore they were removed in the subsequent analysis (the genotyping call rate was 99.5%.). The genotypes of the rs7566605 SNP in individuals from the Multicenter population were obtained by using the commercial Affymetrix GeneChip^® ^Human Mapping 500K Array Set (Affymetrix Inc., CA, USA). The genotyping failed in 6 individuals, and they were also removed in the subsequent analysis (The genotyping call rate was 99.0%.).

### Statistical analyses

χ^2 ^tests were used to determine whether the variant was in equilibrium at its locus in the population (Hardy-Weinberg equilibrium). The effects of the genetic polymorphism on obesity-related measures and cholesterol metabolic traits were assessed by an ANOVA for three genotypes or by a T-test for the dominant and recessive models. Meta-analysis was performed to combine statistical information from independent data sets. The results of the meta-analysis are considered more powerful estimates of the true effect size than those of each study. Bonferroni correction for multiple comparisons was also performed to remove any false positives. All statistical analyses were performed using SAS, version 8.02 (SAS, USA) and Matlab, Version 7.6 (MathWorks, Natick, MA).

## Results

### Association analyses with obesity-related measures

Replication analyses were performed for the association of SNP rs7566605 (located 10 kb upstream of the *INSIG2 *transcription start site) with BMI in three Korean populations (total *n *= 2,634). The allele of rs7566605 was in Hardy-Weinberg equilibrium in all three populations (*P *= 0.415 for Kirin; *P *= 0.911 for Yeungnam; *P *= 0.806 for Multicenter). The each MAF of the SNP in three populations was very similar to the frequency (0.37) for the white population [1] (0.35 for Kirin and Yeungnam populations and 0.33 for Multicenter population; 0.37 for white population).

Association of the rs7566605 genotype with BMI was not found in all three populations (Table 2). When association analyses were extended to include other phenotypes in relation to the considered body features (in this case the WHR, fat mass, fat percent, total AFA, and subcutaneous and visceral AFA), no associations were found for Koreans (Table 3). When the population was divided into two subgroups according to gender (male and female), no associations were shown in either subgroup (Table 2 and Table 3). Associations with obesity-related traits were also not found after the meta-analysis with three populations (data not shown). Therefore, the association between rs7566605 and BMI (as well as other obesity-related measures) was not suitably replicated in Koreans.

**Table 2:** Association analyses of *INSIG2 *rs7566605 with BMI

			**n (mean ± SD)**		
			
**Population**	**Group**	**MAF**	**G/G**	**G/C**	**C/C**
Kirin	Total	0.35	428(26.5 ± 4.61)	432(26.3 ± 4.74)	131(26.1 ± 4.88)
			*P*_*ANOVA *_= 0.689, *P*_*dom *_= 0.423, *P*_*rec *_= 0.564		
	Female	0.35	400(26.1 ± 4.41)	400(25.7 ± 3.96)	120(25.8 ± 4.82)
			*P*_*ANOVA *_= 0.311, *P*_*dom *_= 0.137, *P*_*rec *_= 0.864		
	Male	0.38	28(32.0 ± 3.98)	32(34.2 ± 6.36)	11(29.7 ± 4.79)
			*P*_*ANOVA *_= 0.040, *P*_*dom *_= 0. 405, *P*_*rec *_= 0.0525		
Yeungnam	Total	0.35	312(27.0 ± 3.35)	329(27.2 ± 3.57)	93(27.0 ± 3.26)
			*P*_*ANOVA *_= 0.824, *P*_*dom *_= 0.751, *P*_*rec *_= 0.789		
	Female	0.34	121(26.1 ± 3.80)	119(26.8 ± 4.06)	34(26.3 ± 3.92)
			*P*_*ANOVA *_= 0.378, *P*_*dom *_= 0.218, *P*_*rec *_= 0.837		
	Male	0.36	191(27.5 ± 2.93)	210(27.3 ± 3.26)	59(27.3 ± 2.78)
			*P*_*ANOVA *_= 0.765, *P*_*dom *_= 0.464, *P*_*rec *_= 0.835		
Multicenter	Total	0.33	272(23.1 ± 3.31)	287(22.7 ± 2.99)	66(23.5 ± 3.24)
			*P*_*ANOVA *_= 0.161, *P*_*dom *_= 0.461, *P*_*rec *_= 0.145		
	Female	0.35	168(22.9 ± 3.41)	183(22.3 ± 3.03)	47(23.4 ± 3.29)
			*P*_*ANOVA *_= 0.066, *P*_*dom *_= 0.234, *P*_*rec *_= 0.127		
	Male	0.31	104(23.2 ± 3.15)	104(23.4 ± 2.81)	19(23.7 ± 3.18)
			*P*_*ANOVA *_= 0.790, *P*_*dom *_= 0.581, *P*_*rec *_= 0.585		

The *P *values for BMI in subjects with minor alleles were assessed by an ANOVA for a difference among three genotypes or by a T-test for the dominant and recessive models. The significant threshold was 0.0015 for the Kirin population (11 phenotypes and 3 different models) and 0.0033 for the Yeungnam and Multicenter populations (5 phenotypes and 3 different models).

**Table 3:** Association analysis of *INSIG2 *rs7566605 with the body features of the Kirin population

			**n (mean ± SD)**		
			
**Group**	**Phenotype**	**MAF**	**G/G**	**G/C**	**C/C**
Total	WHR	0.35	428(0.888 ± 0.0728)	432(0.885 ± 0.0776)	131(0.887 ± 0.0811)
			*P*_*ANOVA *_= 0.825, *P*_*dom *_= 0.554, *P*_*rec *_= 0.977		
	Fat mass	0.35	428(24.4 ± 8.36)	432(23.8 ± 8.77)	131(23.8 ± 8.67)
			*P*_*ANOVA *_= 0.590, *P*_*dom *_= 0.305, *P*_*rec *_= 0.689		
	Fat percent	0.35	428(34.3 ± 5.86)	432(33.9 ± 6.13)	131(34.2 ± 6.58)
			*P*_*ANOVA *_= 0.526, *P*_*dom *_= 0.318, *P*_*rec *_= 0.869		
	Total AFA	0.35	422(293 ± 123)	430(291 ± 131)	130(280 ± 122)
			*P*_*ANOVA *_= 0.618, *P*_*dom *_= 0.571, *P*_*rec *_= 0.344		
	Visceral AFA	0.35	422(65.7 ± 43.4)	430(65.5 ± 47.1)	130(60.2 ± 40.3)
			*P*_*ANOVA *_= 0.439, *P*_*dom *_= 0.626, *P*_*rec *_= 0.164		
	Subcutaneous AFA	0.35	422(228 ± 98.0)	430(225 ± 103)	130(220 ± 101)
			*P*_*ANOVA *_= 0.758, *P*_*dom *_= 0.569, *P*_*rec *_= 0.519		
Female	WHR	0.35	400(0.882 ± 0.0697)	400(0.875 ± 0.0664)	120(0.881 ± 0.0798)
			*P*_*ANOVA *_= 0.293, *P*_*dom *_= 0.175, *P*_*rec *_= 0.782		
	Fat mass	0.35	400(23.9 ± 8.17)	400(22.9 ± 7.65)	120(23.6 ± 8.64)
			*P*_*ANOVA *_= 0.228, *P*_*dom *_= 0.123, *P*_*rec *_= 0.845		
	Fat percent	0.35	400(34.5 ± 5.89)	400(33.9 ± 6.14)	120(34.5 ± 6.62)
			*P*_*ANOVA *_= 0.332, *P*_*dom *_= 0.261, *P*_*rec *_= 0.576		
	Total AFA	0.35	394(285 ± 119)	398(276 ± 118)	119(277 ± 121)
			*P*_*ANOVA *_= 0.544, *P*_*dom *_= 0.270, *P*_*rec *_= 0.742		
	Visceral AFA	0.35	394(607 ± 369)	398(591 ± 37.8)	119(556 ± 32.9)
			*P*_*ANOVA *_= 0.399, *P*_*dom *_= 0.313, *P*_*rec *_= 0.192		
	Subcutaneous AFA	0.35	394(225 ± 97.0)	398(217 ± 95.0)	119(221 ± 102)
			*P*_*ANOVA *_= 0.533, *P*_*dom *_= 0.295, *P*_*rec *_= 0.982		
Male	WHR	0.38	28(0.976 ± 0.0598)	32(1.02 ± 0.0891)	11(0.954 ± 0.0656)
			*P*_*ANOVA *_= 0.0292, *P*_*dom *_= 0.192, *P*_*rec *_= 0.0923		
	Fat mass	0.38	28(31.2 ± 8.18)	32(35.0 ± 13.4)	11(26.1 ± 9.06)
			*P*_*ANOVA *_= 0.0629, *P*_*dom *_= 0.589, *P*_*rec *_= 0.0510		
	Fat percent	0.38	28(32.4 ± 5.12)	32(33.9 ± 6.19)	11(30.9 ± 5.23)
			*P*_*ANOVA *_= 0.730, *P*_*dom *_= 0.607, *P*_*rec *_= 0.207		
	Total AFA	0.38	28(406 ± 128)	32(473 ± 146)	11(323 ± 136)
			*P*_*ANOVA *_= 0.00778, *P*_*dom *_= 0.422, *P*_*rec *_= 0.0120		
	Visceral AFA	0.38	28(135 ± 645)	32(146 ± 726)	11(110 ± 72.0)
			*P*_*ANOVA *_= 0.345, *P*_*dom *_= 0.928, *P*_*rec *_= 0.213		
	Subcutaneous AFA	0.38	28(271 ± 103)	32(327 ± 139)	11(213 ± 94.3)
			*P*_*ANOVA *_= 0.0199, *P*_*dom *_= 0.347, *P*_*rec *_= 0.0311		

The *P *values for the AFA in subjects with minor alleles were assessed by an ANOVA for three genotypes or by a T-test for the dominant and recessive models. The significant threshold was 0.0015 for the Kirin population (11 phenotypes and 3 different models).

### Association analyses with cholesterol-related phenotypes and glucose

**Figure 1:**
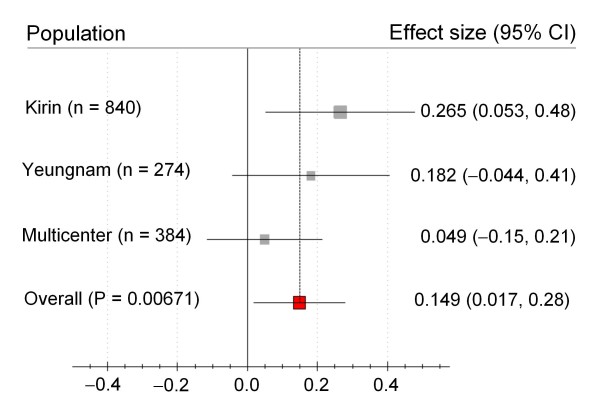
**The pooled effect sizes with 95% CIs for a total cholesterol level in female subgroup**. When the meta-analysis was performed on the three populations, it was found that the rs7566605 SNP was associated with the total cholesterol level in a dominant model, especially in the female subgroup. After performing a Bonferroni correction, a significant effect could not be found on the plasma levels of the total cholesterol (significant threshold: P < 0.0033).

An association analysis with the plasma levels of glucose and cholesterol-related phenotypes (i.e., total cholesterol, HDL cholesterol, and triglyceride) was performed. No genetic effects of the SNP were detected with the levels of glucose, HDL cholesterol, and triglyceride. Among the three populations, the rs7566605 C allele only appeared to be associated with an increased level of total cholesterol in a dominant genetic model in the Kirin population (Table 4). When the association was analyzed for subgroups divided by gender, the genetic effects of the rs7566605 seemed to be maintained in female subjects (Table 4). When the meta-analysis was performed on the three populations (Kirin, Yeungnam, and Multicenter), the rs7566605 C allele was associated in the female subjects with an increased level of total cholesterol (Figure 1). After performing a correction for multiple comparisons with the Bonferroni correction, a significant effect could not be found on the plasma levels of the total cholesterol in female subgroups. However, a marginal association was detected in the total cholesterol levels.

**Table 4:** Association analysis of *INSIG2 *rs7566605 with the levels of cholesterol and triglyceride

			**n (mean ± SD)**		
			
**Group**	**Phenotype**	**MAF**	**G/G**	**G/C**	**C/C**
**Kirin**					
Total	Total cholesterol	0.35	389(4.76 ± 1.42)	395(5.12 ± 1.77)	122(4.76 ± 1.36)
			*P*_*ANOVA *_= 0.00250, *P*_*dom *_= 0.0134, *P*_*rec *_= 0.155		
	HDL cholesterol	0.35	341(1.61 ± 0.907)	328(1.64 ± 0.970)	111(1.42 ± 0.701)
			*P*_*ANOVA *_= 0.0969, *P*_*dom *_= 0.764, *P*_*rec *_= 0.0352		
	Triglyceride	0.35	394(1.03 ± 0.567)	398(0.968 ± 0.597)	122(1.09 ± 0.708)
			*P*_*ANOVA *_= 0.0415, *P*_*dom *_= 0.117, *P*_*rec *_= 0.186		
Female	Total cholesterol	0.35	362(4.78 ± 1.44)	367(5.14 ± 1.78)	111(4.74 ± 1.34)
			*P*_*ANOVA *_= 0.00410, *P*_*dom *_= 0.0170, *P*_*rec *_= 0.178		
	HDL cholesterol	0.35	314(1.64 ± 0.904)	300(1.61 ± 0.896)	100(1.41 ± 0.618)
			*P*_*ANOVA *_= 0.0647, *P*_*dom *_= 0.265, *P*_*rec *_= 0.0203		
	Triglyceride	0.35	368(0.996 ± 0.537)	370(0.910 ± 0.455)	111(1.02 ± 0.613)
			*P*_*ANOVA *_= 0.0396, *P*_*dom *_= 0.0877, *P*_*rec *_= 0.235		
Male	Total cholesterol	0.38	27(4.54 ± 1.10)	28(4.89 ± 1.65)	11(4.56 ± 1.47)
			*P*_*ANOVA *_= 0.629, *P*_*dom *_= 0.480, *P*_*rec *_= 0.728		
	HDL cholesterol	0.39	27(1.30 ± 0.891)	28(1.95 ± 1.54)	11(1.62 ± 1.30)
			*P*_*ANOVA *_= 0.168, *P*_*dom *_= 0.0804, *P*_*rec *_= 0.984		
	Triglyceride	0.38	26(1.57 ± 0.708)	28(1.73 ± 1.33)	11(1.67 ± 1.26)
			*P*_*ANOVA *_= 0.892, *P*_*dom *_= 0.716, *P*_*rec *_= 0.875		
**Yeungnam**					
Total	Total cholesterol	0.35	312 (5.35 ± 0.913)	329 (5.37 ± 0.986)	93(5.39 ± 1.03)
			*P*_*ANOVA *_= 0.930, *P*_*dom *_= 0.751, *P*_*rec *_= 0.760		
	HDL cholesterol	0.35	312 (1.36 ± 0.336)	329 (1.38 ± 0.360)	93(1.37 ± 0.333)
			*P*_*ANOVA *_= 0.771, *P*_*dom *_= 0.509, *P*_*rec *_= 0.954		
	Triglyceride	0.35	312 (1.83 ± 1.33)	329 (1.81 ± 1.54)	93(1.75 ± 0.996)
			*P*_*ANOVA *_= 0.802, *P*_*dom *_= 0.675, *P*_*rec *_= 0.726		
Female	Total cholesterol	0.34	121(5.22 ± 0.855)	119(5.39 ± 1.04)	34(5.44 ± 1.09)
			*P*_*ANOVA *_= 0.296, *P*_*dom *_= 0.124, *P*_*rec *_= 0.445		
	HDL cholesterol	0.34	121(1.49 ± 0.343)	119(1.47 ± 0.357)	34(1.51 ± 0.323)
			*P*_*ANOVA *_= 0.813, *P*_*dom *_= 0.755, *P*_*rec *_= 0.670		
	Triglyceride	0.34	121(1.46 ± 1.20)	119(1.37 ± 0.883)	34(1.38 ± 0.609)
			*P*_*ANOVA *_= 0.784, *P*_*dom *_= 0.610, *P*_*rec *_= 0.781		
Male	Total cholesterol	0.36	191 (5.43 ± 0.943)	210 (5.35 ± 0.956)	59 (5.36 ± 1.01)
			*P*_*ANOVA *_= 0.703, *P*_*dom *_= 0.402, *P*_*rec *_= 0.833		
	HDL cholesterol	0.36	191 (1.28 ± 0.307)	210 (1.33 ± 0.353)	59 (1.29 ± 0.316)
			*P*_*ANOVA *_= 0.275, *P*_*dom *_= 0.173, *P*_*rec *_= 0.716		
	Triglyceride	0.36	191 (2.06 ± 1.36)	210 (2.06 ± 1.77)	59 (1.96 ± 1.11)
			*P*_*ANOVA *_= 0.857, *P*_*dom *_= 0.664, *P*_*rec *_= 0.850		
**Multicenter**					
Total	Total cholesterol	0.34	258(4.80 ± 0.818)	277(4.80 ± 0.804)	63(4.97 ± 0.798)
			*P*_*ANOVA *_= 0.273, *P*_*dom *_= 0.660, *P*_*rec *_= 0.107		
	HDL cholesterol	0.34	258(0.884 ± 0.818)	277(1.15 ± 0.293)	64(1.19 ± 0.325)
			*P*_*ANOVA *_= 0.870, *P*_*dom *_= 0.625, *P*_*rec *_= 0.965		
	Triglyceride	0.34	258(1.31 ± 0.949)	277(1.35 ± 0.904)	63(1.15 ± 0.639)
			*P*_*ANOVA *_= 0.288, *P*_*dom *_= 0.988, *P*_*rec *_= 0.133		
Female	Total cholesterol	0.35	159(4.81 ± 0.798)	179(4.81 ± 0.819)	46(5.03 ± 0.780)
			*P*_*ANOVA *_= 0.216, *P*_*dom *_= 0.560, *P*_*rec *_= 0.0801		
	HDL cholesterol	0.35	159(0.883 ± 0.0539)	179(0.883 ± 0.0972)	47(0.879 ± 0.0161)
			*P*_*ANOVA *_= 0.943, *P*_*dom *_= 0.894, *P*_*rec *_= 0.733		
	Triglyceride	0.35	159(1.18 ± 0.764)	179(1.12 ± 0.646)	46(0.99 ± 0.429)
			*P*_*ANOVA *_= 0.244, *P*_*dom *_= 0.214, *P*_*rec *_= 0.144		
Male	Total cholesterol	0.31	99(4.79 ± 0.852)	98(4.77 ± 0.779)	17(4.81 ± 0.845)
			*P*_*ANOVA *_= 0.978, *P*_*dom *_= 0.916, *P*_*rec *_= 0.883		
	HDL cholesterol	0.31	99(0.884 ± 0.0559)	98(0.876 ± 0.0185)	17(0.892 ± 0.102)
			*P*_*ANOVA *_= 0.336, *P*_*dom *_= 0.389, *P*_*rec *_= 0.359		
	Triglyceride	0.31	99(1.52 ± 1.16)	98(1.77 ± 1.13)	17(1.57 ± 0.895)
			*P*_*ANOVA *_= 0.300, *P*_*dom *_= 0.157, *P*_*rec *_= 0.817		

The *P *values for the levels of cholesterol and triglyceride in subjects with minor alleles were assessed by an ANOVA for a difference among three genotypes or by a T-test for the dominant and recessive models. The significant threshold was 0.0015 for the Kirin population (11 phenotypes and 3 different models) and 0.0033 for the Yeungnam and Multicenter populations (5 phenotypes and 3 different models).

## Discussion

*INSIG2*, showing involvement with cholesterol metabolism in studies using the cell line and mice, has been identified as an obesity-susceptible gene in a recent whole-genome association study [1]. However, a number of contrasting reports exist regarding the genetic effect of the gene polymorphism (rs7566605) on obesity. The association with BMI also could not be replicated in the present research with Korean populations. The proposed relationship shown in mice between *INSIG2 *and cholesterol metabolism has not been verified in human studies of white [8,12,13], African [12], Chinese [15], Japanese [4], and Uyghur [6] populations. Our results demonstrated that the C allele of *INSIG2 *rs7566605 did not significantly affect the cholesterol levels.

However, a possible relationship was found between the *INSIG2 *and the plasma level of total cholesterol in females (Table 4). From a high-density SNP analysis in mice, *Insig2 *was identified as a susceptible gene in the control of plasma cholesterol levels [19]. *Insig2 *has been strongly associated with cholesterol biosynthetic genes that can be activated by *Srebp-2 *through construction of a transcriptional network in the female liver. This implies that the carriage of the rs7566605 C allele might weaken the blocking of SREBP activation, leading to elevated total cholesterol levels. However, a relationship between rs7566605 and the cholesterol level could not be established in the present work because there were no significant associations in the meta-analysis and the mean level of the total cholesterol was higher for heterozygotes than those of the major and minor homozygotes in the Kirin population. Therefore, it is necessary to perform an association analysis with other large populations and/or to do research on the subjects who are restricted to having a cholesterol-rich diet during a definite period in order to elucidate the relationship and conclude whether INSIG2 is related with cholesterol metabolism in humans.

Whether INSIG2 plays a role in obesity development in Korean subjects remains unclear, as a relationship between rs7566605 and BMI was not found. However, an association with obesity has been reported in many studies with white, Asian, and African populations [1-6,26]. According to the results of Cervino et al. [19], *INSIG2 *might be related to an increase in fat mass, as *Insig2 *was identified upstream of several obesity-related genes in the transcriptional network. This can be supported by a study with double-knockout mice in relation to both *Insig1 *and *Insig2*, where mice given a cholesterol-rich diet gained more weight compared to a control group. From a recent study, a new polymorphism, *INSIG2 *-102G/A, in high linkage disequilibrium with rs7566605 (D' = 0.96 and r^2 ^= 0.04) was found to be related to an increase in BMI and to adipogenesis through SREBP1 activation. However, no connection was found with triglyceride, cholesterol, or glucose [20]. The -102G/A polymorphism has been proposed as the functional polymorphism of *INSIG2*, as it appears to influence the level of *INSIG2 *expression directly though the weaker binding of nuclear factors on the -102A DNA fragment. Therefore, the genetic effects of the *INSIG2 *gene on fat mass accumulation can be clarified by further studies with the new putative functional variant, and the effects may be strengthened by the habitual ingestion of cholesterol-rich food.

## Conclusion

The present study indicates there is no significant relationship between the *INSIG2 *gene and cholesterol levels, although the results indicate that the *INSIG2 *gene tended to affect the total cholesterol level that was obtained in this study. From human epidemiological studies conducted to date, the influence of *INSIG2 *on body fat accumulation and cholesterol metabolism is unclear. Therefore, it is necessary to determine the roles of *INSIG2 *in the creation of body fat and in cholesterol metabolism, and to elucidate the interplay among INSIG1 (another INSIG protein), INSIG2, and the SREBP pathway on the two metabolisms.

## Abbreviations

AFA: abdominal fat area; ANOVA: analysis of variance; BMI: body mass index; 95% CI: confidence interval at 95%; HDL cholesterol: high-density lipoprotein cholesterol; INSIG1: insulin-induced gene 1; INSIG2: insulin-induced gene 2; MAF: minor allele frequency; PCR: polymerase chain reaction; SCAP: SREBP cleavage-activating protein; SD: standard deviation; SNP: single nucleotide polymorphism; SREBP: sterol regulatory element-binding protein; WHR: waist-to-hip ratio.

## Competing interests

The authors declare that they have no competing interests.

## Authors' contributions

CS designed the study, interpreted the data, and wrote the manuscript. IK performed the statistical analyses and interpretation. SMC recruited subjects and managed the clinical data. BLP assisted with study design, and directed the genotyping analyses. KSK recruited subjects and performed clinical measurements. JRK recruited subjects and performed clinical measurements. HDS participated in the study design, and involved in drafting the manuscript. JYK supervised the study by overseeing the recruitment of subjects and involved in drafting the manuscript. All authors read and approved the final manuscript.

## Pre-publication history

The pre-publication history for this paper can be accessed here:


